# Editorial: Variability and Individual Differences in Early Social Perception and Social Cognition

**DOI:** 10.3389/fpsyg.2016.00068

**Published:** 2016-02-03

**Authors:** Alia Martin, Talee Ziv, Jessica A. Sommerville

**Affiliations:** ^1^Department of Psychology, Harvard UniversityCambridge, MA, USA; ^2^Department of Psychology, University of WashingtonSeattle, WA, USA

**Keywords:** infant cognition, developmental mechanisms, individual differences, cognitive development, social cognition, social perception

In this research topic, we showcase state-of-the-art research on the sources and meaning of variability and individual differences in early social perception and cognition. These papers demonstrate that such variability contributes to our understanding of early development, requires specific methodological toolboxes and skill sets to expand our developmental inventory, and relates to important abilities later in life.

The first set of papers (Examining individual variability to elucidate developmental mechanisms and pathways) highlights the importance of focusing on individual variability (above and beyond group variability) for understanding the underlying mechanisms of early social cognition and how meaningful early differences relate to abilities later in childhood. !http://dx.doi.org/10.3389/fpsyg.2015.00858!!Hepach et al.!!! propose that studies of social cognition go beyond using a single behavioral measure (i.e., looking time) and offer two novel measures, pupil dilation and postural changes. They argue that these measures may provide insight into the mechanisms and motivations underlying behavioral responses (e.g., prosocial behaviors) in which there may be individual differences even if behavioral indices are similar at a group level.

The other papers in this section use longitudinal research to investigate the meaning of measures of infant social cognitive development in the context of broader developmental trajectories. !http://dx.doi.org/10.3389/fpsyg.2015.00327!!Gampe et al.!!! found that infants' action perception and action production in a contralateral reach task improve consistently across the second half of the first year of life at a group level; however, infants' individual action production abilities were not correlated across time, and there was no consistent causal relation between action perception and production across age. Thus, this study of individual differences uncovers a dynamic link between action perception and production in the first year of life. !http://dx.doi.org/10.3389/fpsyg.2015.00719!!Brink et al.!!! investigate how infant social attention relates to preschool social cognition. They found that infants' habituation to a social display of intentional action was related to measures of infant social-interactive experiences and temperament and, later, preschool theory of mind. These studies highlight the role of both experience and temperament in predicting social cognitive abilities, as each factor was an independent predictor of preschool theory of mind ability. Adding to this work on the downstream consequences of social cognition in infancy, !http://dx.doi.org/10.3389/fpsyg.2015.00789!!Kristen-Antonow et al.!!! examine effects of early individual differences in social cognition on the developing sense of self. Infants' social responsiveness in a still-face paradigm predicted their later mirror-self-recognition, and their imitation abilities predicted later delayed-self-recognition. This work emphasizes the importance of measuring variability to expose the underlying mechanisms of development.

The next three sets of papers focus on variability and individual differences in early social cognition across several other topics. For example, while it is known that infants attend to and encode others' goal-directed actions from an early age, the papers by !http://dx.doi.org/10.3389/fpsyg.2015.00059!!Bakker et al.!!!, !http://dx.doi.org/10.3389/fpsyg.2015.00310!!Gerson et al.!!!, and !http://dx.doi.org/10.3389/fpsyg.2015.01487!!Dunfield and Johnson!!! illustrate that there are subtle nuances in how different individuals might process goal-related events (Variability in the encoding of actions and goals). !http://dx.doi.org/10.3389/fpsyg.2015.00059!!Bakker et al.!!! report an EEG study with 9-month-old infants. They found differences in the P400 component across their sample when infants observed a typical instance of intentional non-verbal communication, a “give-me” gesture, vs. a similar non-intentional gesture, a rotated hand. However, the P400 differences between the two types of gesture were significantly larger in female infants than male infants, suggesting a deeper encoding of communicative intent in females and raising questions about what mechanisms might lead to these differences.

!http://dx.doi.org/10.3389/fpsyg.2015.00310!!Gerson et al.!!! also show evidence of individual differences in 8-month-old infants' goal processing. Infants who became more planful in their goal-directed actions after training in a means-end task were more able to process another person's goal in a means-end task than infants who received the training but did not become as planful afterward. These studies suggest that infants' own goal-directed action production not only influences their ability to parse goal-directed behavior, but that this link varies based on infants' success in learning to achieve their goals.

!http://dx.doi.org/10.3389/fpsyg.2015.01487!!Dunfield and Johnson!!! ask whether instrumental and social goals differ, and consider whether the study of adult social cognition can inform the study of infant social cognition. When goals were unambiguous, they found no individual differences in adults' ability to identify instrumental goals or social goals. When goals were ambiguous, however, insecurely attached adults were significantly less likely to attribute social goals than securely attached adults. All of these papers suggest that there is meaningful variability in how we perceive and interpret goals across development.

The next set of papers focuses on variability in how infants perceive the facial and affective information in their environment (Variability in the factors moderating children's encoding of facial and affective information). !http://dx.doi.org/10.3389/fpsyg.2015.01330!!Kim et al.!!! report a preference for female faces over male faces in 3- and 10-month-old infants, but only for white faces (not black or Asian). This effect did not differ based on the race of infants' own primary caregiver. Interestingly, !http://dx.doi.org/10.3389/fpsyg.2015.00593!!Liu et al.'s!!! study conducted in China with Chinese infants revealed a preference for female over male Asian faces at 3 and 6 months, but not 9 months of age. Furthermore, this gender preference was not observed for Caucasian faces, highlighting the importance of examining variability across cultural contexts. !http://dx.doi.org/10.3389/fpsyg.2015.00593!!Liu et al.!!! also found an effect of experience on the own-race female face preference in their sample: infants who had more experience with male caregiving showed a decreased preference for female faces.

!http://dx.doi.org/10.3389/fpsyg.2015.00922!!Ravicz et al.!!! ask whether temperament differences in infants are related to affective perception in the form of responses to emotional facial expressions. Using fNIRS, they tested infants' neural responses to happy female faces, and found that infants with lower negative emotionality showed more preferential left hemisphere prefrontal cortex activation to the happy facial expressions. !http://dx.doi.org/10.3389/fpsyg.2015.00846!!Ben-Israel et al.!!! examine how innate factors may influence affective processing. They investigated whether a dopamine D4 receptor polymorphism is related to sex differences in affective knowledge in young children. In a longitudinal twin study, they found that for carriers of the 7-repeat allele in both their 3- and 5-year-old samples, boys had higher affective knowledge scores than girls, but there were no sex differences in non-carriers. These differences were in contrast to sex differences found in adults in which females with the polymorphism show increased empathy compared to males. These results illustrate the importance of considering that variability (for example, in genetic effects on cognition) may differ in type and meaning at different developmental timepoints.

In the following section (Effects of variability in linguistic experience and ability on early cognition), two papers examine the effects of variation in early linguistic experience and skills on children's social cognitive abilities. !http://dx.doi.org/10.3389/fpsyg.2015.00561!!Zimmermann et al.!!! found the typical video transfer deficit in a puzzle-solving task: 2-year-olds solved the puzzle more effectively following imitation of a live model compared to a non-interactive video model. Yet individual differences in children's ability to spontaneously generate a semantic label for the puzzle (e.g., “a sailboat”) were associated with a reduction in the video transfer deficit, suggesting that children's ability to solve the problem using a verbal cue helped them to learn from a non-interactive model.

!http://dx.doi.org/10.3389/fpsyg.2015.00332!!Henderson and Scott!!! focus on the role of linguistic experience on infants' understanding of language as a conventional system, building on previous work showing that by 9 months monolingual infants expect two speakers to use the same word to refer to an object. They found that variability in language exposure has an important influence on this expectation. Thirteen-month-olds growing up in a bilingual environment did not expect two speakers who speak the same language to use the same word to refer to an object, suggesting that bilingual infants may have expectations of conventionality in language that are different from those of monolingual infants.

The final group of papers (Relation of parent and socialization factors to early individual variability in social cognition) brings up an important source of variability in early experience that can affect social perception and cognition: parent socialization. !http://dx.doi.org/10.3389/fpsyg.2015.00360!!Upshaw et al.!!! measured 12- and 15-month-old infants' pupil dilation as an index of arousal in response to viewing displays of infants expressing happy and sad emotion. They found that participants' arousal was meaningfully associated with their parents' social dispositions and behaviors. Infants' arousal in response to sad emotion was correlated with parent self-reported empathic perspective taking, and infants' arousal to happy emotion was correlated with parent self-reported altruism. !http://dx.doi.org/10.3389/fpsyg.2015.00600!!Gross et al.!!! conducted a complementary study which directly assessed parental reports of their infant socialization practices and looked at relationships to infant social attention and prosocial behavior. Overall, parent socialization of prosocial behavior was related to some aspects of prosocial behavior, and moderated the link between social understanding and prosocial behavior. Interestingly, parent socialization mattered more than infant temperament, which did not meaningfully predict differences in prosocial behavior. Finally, !http://dx.doi.org/10.3389/fpsyg.2015.00354!!Wade et al.!!! show that the way parents socially interact with their infants can serve as a protective factor against potential risks to infant social cognitive development. In a longitudinal study, they found that cumulative biomedical risk affects variability in social cognition at 18 months, but that this relationship is moderated by parental responsiveness; that is, higher biomedical risk predicted reduced social cognitive abilities only for infants with less responsive parenting.

Taken together, the papers in this Research Topic underscore the importance of considering variability and individual differences in infants' early social perception and cognition, seeking out appropriate methods to accurately measure such variability, and looking for meaningful sources of these differences that can shed light on the mechanisms underlying children's early abilities as well as developmental change over time.

## Author contributions

All authors (AM, TZ, JS) contributed equally to the editing process and organization of the Research Topic. AM drafted the editorial, and TZ and JS provided critical feedback and edits.

### Conflict of interest statement

The authors declare that the research was conducted in the absence of any commercial or financial relationships that could be construed as a potential conflict of interest.

